# Microplastics as a Threat to Aquatic Ecosystems and Human Health

**DOI:** 10.3390/toxics12080571

**Published:** 2024-08-05

**Authors:** Agata Witczak, Laura Przedpełska, Kamila Pokorska-Niewiada, Jacek Cybulski

**Affiliations:** Department of Toxicology, Dairy Technology and Food Storage, Faculty of Food Sciences and Fisheries, West Pomeranian University of Technology in Szczecin, 70-310 Szczecin, Poland; laura_przedpelska@o2.pl (L.P.); kamila.pokorska@zut.edu.pl (K.P.-N.); jacek.cybulski@zut.edu.pl (J.C.)

**Keywords:** microplastics in aquatic environments, adsorption of contaminants, degradation of microplastics, impact on human health

## Abstract

The threat posed by microplastics has become one of the world’s most serious problems. Recent reports indicate that the presence of microplastics has been documented not only in coastal areas and beaches, but also in water reservoirs, from which they enter the bodies of aquatic animals and humans. Microplastics can also bioaccumulate contaminants that lead to serious damage to aquatic ecosystems. The lack of comprehensive data makes it challenging to ascertain the potential consequences of acute and chronic exposure, particularly for future generations. It is crucial to acknowledge that there is still a substantial need for rapid and effective techniques to identify microplastic particles for precise evaluation. Additionally, implementing legal regulations, limiting plastic production, and developing biodegradation methods are promising solutions, the implementation of which could limit the spread of toxic microplastics.

## 1. Introduction

**Figure 1:**
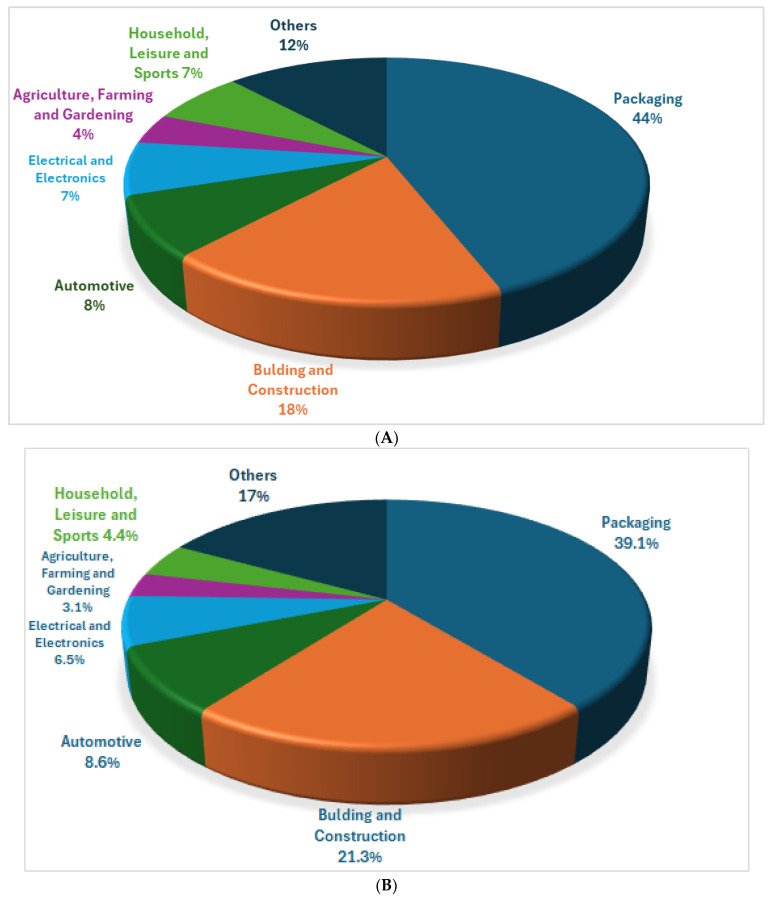
(**A**) Use of plastics according to economic sector globally in 2021 based on [4]. (**B**) Use of plastics according to economic sector in Europe in 2021 based on [4].

Plastics are a group of materials whose most significant component is a polymer. Polymers are the foundation of plastic and are used in a wide variety of applications, including packaging, automotive, construction, electronics, medical devices, and the textile industry [1]. Low- and high-density polyethylenes (LDPE, HDPE), polypropylene (PP), polyvinyl chloride (PVC), polystyrenes (PS, EPS), polyethylene terephthalate (PET), and polyurethanes (PUs) are the largest contributors to the industry [2,3]. Because of their widespread use, they are of great importance to many sectors of the economy and are therefore difficult to reduce or eliminate. The Plastics Europe report [4] indicates that the largest global demand for plastics is from the packaging (44%), construction (18%), and automotive (8%) industries. The use of plastics in 2021 according to economic sector is given in Figure 1A,B. According to Plastics Europe [5], global plastics production exceeded 400 million tons in 2022, with half of it comprising single-use plastics [6]. Carpenter and Smith [7] were the first to publish in the scientific literature on the problem of microplastics after discovering plastic particles off the coast of the North Atlantic Ocean. Microplastics are pollutants that are created by the improper management of waste from plastic production. In 2016, at the World Economic Forum [8], the magnitude of the problem with regard to aquatic basins was highlighted, as they contain more than 150 million tons of plastic waste. The threat stemming from the occurrence of microplastics has become a global problem because of their ubiquitous bioavailability in the environment and the effects they have not only on aquatic organisms but also on humans. Further, microplastics can also bioaccumulate contaminants that lead to serious damage to aquatic and terrestrial ecosystems [9].

The biodegradation of the most commonly used polymers remains challenging. Polymers such as PE, PP, and PVC are not readily biodegradable; they are subjected to weathering and fragmenting into micro- and nanoplastics and remain in the environment for hundreds of years [6]. Plastics in marine environments are weathered by exposure to sunlight and oxidizing conditions, leading to their slow degradation [10]. Conversely, biodegradable plastics such as polybutylene succinate (PBS), polycaprolactone (PCL), and polyvinyl alcohol (PVA) only undergo complete degradation at temperatures exceeding 50 °C, a condition that is seldom encountered in marine environments [6].

The aim of this article is to review the current state of knowledge on the threat posed to the aquatic environment by the presence of microplastics and their impact on the functioning of aquatic organisms, as well as the risk to human health. It discusses the fate of microplastics in the aquatic environment and the factors influencing their biodegradation, with a particular focus on the use of microorganisms. It also raises the important issue of the adsorption and transfer of pollutants by microplastics.

## 2. Microplastics—Nomenclature, Sources, and Creation of Microplastics

Currently, there is no unified, common definition of microplastics [11]; however, microplastics are recognized as heterogeneous mixtures of materials of various shapes defined as fragments, fibers, spheroids, granules, flakes, and balls in sizes ranging from 0.1 µm to 5 mm, with particles <0.1 µm classified as nanoplastics [12,13]. Microplastics are divided into primary microplastics and secondary microplastics depending on their sources [1,3]. Primary microplastics are plastic pieces that are produced in small sizes for industrial purposes and include powders, granules, and flakes that are added intentionally to products. Sources of primary microplastics in the environment are personal care products, industrial cleaning products, abrasive materials, and synthetic fabrics. Microplastics from personal care, cosmetic, and pharmaceutical products can enter the marine environment with wastewater [14]. They can also originate from the erosion of car tires while driving, or the abrasion of synthetic clothes during washing; other sources include city dust or road markings [15]. The primary source of microfibers in the environment is represented by synthetic fabrics, with emissions estimated to range from 4.3 to 7.0 million tons [16]. When washed, fabrics release microfibers composed of polyester, polyethylene, or acrylic [17]. The deterioration of fabrics is a gradual process caused by, inter alia, wear, flexing, and abrasion. These factors can accelerate the unraveling of threads and the formation of unspun microfibers [18]. Secondary microplastics are not produced intentionally; they are generated by the breakdown of larger-sized plastic debris through physical, sunlight radiation, and biological degradation processes [9,19]. Factors that contribute to the degradation of polymers into secondary microplastics include ultraviolet radiation, changes in temperature, and physical abrasion caused by the movements of water, sand, and wind [20]. They also form because of microbiological and biological degradation by marine organisms. One of the basic mechanisms that lead to the formation of secondary microplastics is induced photodegradation, during which the polymer matrix is oxidized by ultraviolet radiation from sunlight that causes chemical bonds to break. Secondary microplastics often accumulate in the marine and coastal environment due to the degradation of fishing nets and lines from industrial and fishing operations, and plastic bottles and bags caused by intensive tourism [15,19,21,22]. Studies conducted by Wright et al. [23] have demonstrated that the decomposition of ALDFG can result in the generation of 1.277 ± 0.41 pieces of microplastics per meter of beach. However, there is currently a paucity of information regarding the size, number, distribution in the aquatic environment, and microplastic emissions resulting from the decomposition of these materials. Further research is required to address these knowledge gaps.

## 3. The Fate of Microplastics in the Aquatic Environment

The fate of microplastics in the environment and their transport depend on particle density, shape, composition, and size. The movement of microplastics from land to water is influenced by the type of land cover [9,24]. This discrepancy is effectively demonstrated by Luo et al. [25], who indicate that the highest concentration of microplastics in water bodies is situated in areas of agricultural and grassland use. Moreover, microplastic concentration decreases moving away from the shore [26]. In addition, higher levels of contamination by microplastics have been noted in an enclosed bay-type beach compared with open delta-type beaches [27]. Such accumulation of plastic on land results in its entry into water systems, which is exacerbated by inadequate waste disposal systems [14].

The presence of microplastics in water affects not only aquatic organisms, but also the water flow rate, water depth, and bottom topography [10,28]. Plastic microplastics tend to increase downstream, and plastic microplastic pollution is high in estuaries, as observed in the Huangpu River [29]. In contrast, the amount of microplastics in the form of fibers tends to decrease from small water bodies to the sea [30]. The concentrations of microplastics may also decrease due to dilution as they move into the ocean [31]. Microplastic contamination of water also depends on the hydrological and morphological characteristics of given basins, meteorological and climatic phenomena (thunderstorms, storms, precipitation), and socio-economic factors [10,32]. The extent of microplastic pollution in aquatic environments and its impact on reservoir characteristics remain largely unstudied and warrant further research.

Microplastics are distributed across the floor of the ocean, the water column, the seabed, and coastlines and degrade through various biological, physical, and chemical mechanisms [14]. Physical degradation includes phenomena such as weathering, fragmenting, and biofouling, while tides and strong currents also influence transport to marine environments [14]. Chemical degradation includes particle oxidation and hydrolysis [21,24], which have a significant influence on the formation of secondary microplastics. Microorganisms, including bacteria, molds, and algae, play the most important roles in degradation. These organisms degrade microplastics through hydrolysis and enzymatic catalysis [33], during which they secrete, among other things, esterases, ureases, lipases, proteases, glycoside hydrolases, and laccase that attach to the frameworks of long-chain plastic polymers and cleave them into monomer components [34]. During biodegradation, microorganisms obtain the energy necessary for their metabolic transformations. In cells, monomers are mineralized under aerobic conditions into CO_2_ and H_2_O and under anaerobic conditions also into CH_4_, which then produce biomass [35].

Environmental factors such as temperature, pH, humidity, salinity, and material properties determine the ability of microorganisms to adhere and form a biofilm. Liu et al. [36] developed an innovative method of microplastic biodegradation involving the creation of a bacterial biofilm based on the ‘trap and release’ mechanism. Extracellular polymeric substances (EPSs) are sticky colloidal substances produced by biofilms that capture microplastics and bioaggregate particles that are then released in a form suitable for further recycling and processing. Torena et al. [37] found that *Bacillus cereus* and *Agromyces mediolanus* caused 17% degradation of PET. Ren and Ni [38] also obtained microplastic mass losses for PHB, PE, and PHA of 13.4%, 13%, and 12.7%, respectively. Bacteria of the genera *Pseudomonas*, *Pandoraea*, and *Dyella* exhibited activity against these materials within 67 to 116 days. Furthermore, Pikoli et al. [39] identified *Bacillus paramycoides* as the strain that had the highest PS biodegradation capacity, with microplastic mass losses of over 11% within 42 days. Some species of microalgae and fungi are also able to eliminate microplastics. Literature reports indicate, inter alia, that *Chlorella pyrenoidosa* can biodegrade BPA and PS [40], and *Chlorella vulgaris* can biodegrade PET [41]. Using *Zalerion maritimum*, a marine fungus species, to effectively biodegrade PE is also interesting as this species can significantly reduce microplastic mass when small amounts of nutrients are available [42].

### Microplastics in Sediments, Seawater, and Freshwater

The prevalence of processes contributing to the distribution of microplastics in water is illustrated by studies conducted with the aim of determining their contents in aquatic ecosystems. Nava et al. [32] analyzed 38 lakes in different global locations in which plastic wastes occurred, and in more than 93% of the locations analyzed, the wastes were classified as microplastics. Most of the locations analyzed (55%, 21 lakes) had concentrations of less than one particle per m^−3^, 14 (37%) had concentrations between one and five particles per m^−3^, and three (8%) had concentrations higher than five particles per mm^−3^ [32]. The study by Graca et al. [26] indicates a lower concentration of microplastics in the Baltic Sea bottom sediments (0–27 particles/kg d.m.) than in beach sediments (up to 53 particles/kg d.m.), which indicates the concentration of microplastics clearly decreased with distance from the shore to the sea. The distribution and concentration of microplastics in aquatic environments are influenced by the area of their occurrence, as evidenced by Table 1 and Table 2. Table 1 presents the findings from littoral, surface, and beach sediments, while Table 2 illustrates the occurrence of microplastics in surface water samples.

## 4. Occurrence of Microplastics in Aquatic Organisms

The presence of microplastics in water bodies has prompted research with the aim of determining their presence in aquatic organisms. Horton et al. [65] conducted research to evaluate the occurrence of microplastics in the digestive tracts of European flounder (*Platichthys flesus*), whiting (*Merlangius merlangus*), and Atlantic herring (*Clupea harengus*) sampled from the rivers Thames and Stour. In these locations, it was confirmed that 41.5% of the fishes ingested at least one microplastic particle (37.5% of European flounder, 52.2% of whiting, and 28.6% of Atlantic herring) [65]. Hamed et al. [66] studied fish available at markets in Egypt and found no microplastics in either the muscles or livers, but they observed them in stomachs and intestines. Further, Zhu et al. [67] observed microplastics in the gills of fishes and the soft tissues of oysters they examined. Mazlan et al. [68] confirm the occurrence of microplastics identified as polyethylene (PE) and poly(methyl methacrylate) (PMMA) in a species of sea cucumber (*Acaudina molpadioides*). Scott et al. [69] note microplastics in 238 of 269 (88.5%) samples of blue mussel (*Mytilus edulis*) collected at nine locations on the southeastern coasts of Great Britain. Among these particles, 87% were microfibers and 12% were plastic fragments. Provencher et al. [70] conclude that over 690 species of marine organisms ingest microplastics. Reports indicate that concentrations of microplastics are higher in benthic fishes than in pelagic fishes [65,67]. Benthic fishes are more exposed to both accidental and intentional ingestion of microplastics given the habitat they inhabit; in addition, a high amount of contaminants accumulates on the bottom of water basins [66]. A comprehensive overview of the prevalence of microplastics, with a distinction between freshwater and seawater organisms, is presented in Table 3.

### The Impact of Microplastics on the Functioning of Aquatic Organisms

Microplastics are of similar sizes to plankton, which is why aquatic invertebrates can ingest them, causing disruptions in the functioning of their bodies. The problem of the impact of microplastics on the functional characteristics of benthic aquatic organisms can be analyzed according to various criteria, including behavior (motor activity, interactions with substrates, spontaneous movements), energy and metabolism (energy and oxygen use, assimilation effectiveness, macronutrient availability), nutrition (ingestion and excretion rates, predation efficiency, feeding), somatic development (weight, body length and width, growth rates, changes in body weight), mortality, and reproduction [91].

Undigested microplastic particles are often not excreted by aquatic organisms, and they disrupt nutrient assimilation, cause stomach blockages, damage mucus membranes, and cause digestive tract infections that can lead to starvation and even death [92]. Greater quantities of microplastics are found in the stomachs of marine mammals than in the intestines, which indicates that stomachs are potentially a temporary retention site [93]. Microplastics have been shown to be neurotoxic and participate in inhibiting acetylcholinesterase secretion and increased lipid oxidation in muscles and brains [94], which are symptomized in organisms by oxidative and liver disease [93,95]. Further, there are indications that some invertebrates prefer consuming microplastics over their natural foods [94].

When studying the toxicity of microplastics and their impact on the functioning of organisms in the aquatic ecosystem, it is important to consider their quantity and size and the types of polymers that are detected in individual tissues and organs. The toxicological effects of direct ingestion of microplastics by wild animals remain poorly understood. Barboza et al. [96] examined the effects of microplastics on the performance of three species of wild fish: *Dicentrachus labrax*, *Trachurus trachurus*, and *Scomber colias*. Microplastics were found in the digestive tract, gills, and dorsal muscles of 49% of the fish tested, which resulted in significant oxidative damage and symptoms of neurotoxicity. A study by Zitouni et al. [97] on the effects of microplastics on the liver of wild fish (*Serranus scriba*) found notable cytotoxicity, neurotoxicity, and genotoxicity: the fish demonstrated modified acetylcholinesterase activity, changes in malondialdehyde content, and the presence of reactive oxygen species. Cocci et al. [98] demonstrated that the presence of microplastics in the digestive tracts of wild *Mullus barbatus* and *Merluccius merluccius* fish was associated with increased cytokine production, ROS generation, and infiltration of immune cells, which collectively contribute to intestinal inflammation.

The impact of microplastics on living organisms is a highly problematic and complex issue that requires consideration of the natural physiology of organisms, the impact of microplastic toxicity, mechanical damage, and the role of microplastics in organisms exposed to prolonged exposure in laboratory conditions. Zebrafish are the organism of choice in laboratory experiments due to several advantages, including their relatively small size, ease of culture, and relatively short life cycle [99]. Lu et al. [100] showed that zebrafish (*Danio rerio*) exposed to polystyrene microplastics exhibited changes in lipid and energy metabolism. Furthermore, histopathological analysis indicated significant swelling and hepatitis. Feng et al. [101] report that zebrafish embryos exposed to polystyrene nanoplastics demonstrated reduced hatching rate and embryo survival; in addition, nanoplastics inhibited heart rate and resulted in the accumulation of reactive oxygen species. Furthermore, Bashirova et al. [102] examined the mechanism of the toxic effects of PET nanoparticles, which accumulated significantly in the intestines, liver, and kidneys of zebrafish. It was found that the nanoplastic caused oxidative stress, disruption of mitochondrial membrane integrity and phospholipid hydrolysis, and changes in energy metabolism pathways [102].

Another organism studied in the literature is medaka (*Oryzias latipes*). Yu et al. [103] report the mechanism of toxicity associated with polystyrene nanoplastics in medaka involved disruption of cell membrane fluidity, lipid peroxidation, and increased accumulation of contaminants present in the organisms. Furthermore, polystyrene nanoplastics demonstrated chronic hepatotoxic effects leading to changes in liver enzymatic activity and structural damage [104]. Furthermore, exposure to polystyrene nanoplastic resulted in intestinal dysfunction and increased permeability, as well as reduced diversity and composition of the intestinal microflora [105].

Although zebrafish and medaka are the frequently studied animal models in laboratory experiments, there has been a recent shift towards research on organisms other than fish, as evidenced by the research of Jaikumer et al. [20] on crustacean species. The authors studied the impacts of primary and secondary microplastics on the reproduction of three freshwater cladoceran species (*Daphnia magna*, *Daphnia pulex*, *Ceriodaphnia dubia*) during chronic exposure. These researchers concluded that the reproductive capacity of all three species declined during exposure to microplastics, as evidenced by a decrease in brood size. They also observed that primary microplastics were potentially more toxic than secondary microplastics. The no-observed-effect concentration (NOEC) was lower than the lowest concentration studied of 10^2^ p/mL [20]. Laboratory experiments do not reproduce the conditions in the natural environment, but they do allow the effects of acute and chronic exposure to be determined. Furthermore, test organisms are usually exposed to one type of polymer of a well-defined size at high concentrations [106].

The current body of research is insufficient to elucidate the precise mechanism of microplastic toxicity on aquatic organisms. Consequently, there is still a lack of studies revealing the effects of exposure to different types of micro- and nanoplastics, taking into account polymer types, different sizes, shapes, and colors. This is a serious problem not only from the perspective of aquatic ecosystem functioning, but also from the perspective of the health of people who consume food products of aquatic origin.

## 5. Adsorption of Contaminants by Microplastics

Microplastics are characterized by large specific surface areas and low polarity and are highly hydrophobic, which is why they accumulate chemical pollutants easily [2,107] and are reservoirs of such contaminants. The quantities of contaminants collected on the surfaces of microplastics are influenced by environmental conditions, chemical contaminant characteristics, polymer types, and environmental contaminant concentrations. Concentrations of chemical contaminants can be up to six times higher on the surface of microplastics than in the surrounding free water [108].

Above all else, microplastics accumulate or adsorb persistent organic pollutants (POPs), and this increases their toxicity substantially [108]. POPs include, inter alia, polychlorinated biphenyls (PCBs), polycyclic aromatic hydrocarbons (PAHs), polybrominated diphenyl ethers (PBDEs), and dichloro-diphenyl-trichloroethane (DDT). Llorca et al. [109] assessed the adsorption capacity of PCB markers on microplastic surfaces of three polymers—PS, PE, and PET. They demonstrated that microplastics adsorbed 20–60% of the PCBs after three weeks of exposure. Gorman et al. [110] determined the concentrations of PAH and PCB associated with microplastic pellets collected along the South Atlantic coastline and reported that concentrations of PAHs clearly exceeded the threshold level for sediments (1.454–6.002 ng/g) and that PCBs (0.8–104.6 ng/g) were dominated by low-chlorinated congeners likely originating from industrial areas. Herrera et al. [111] evaluated contaminant accumulation in *Dicentrarchus labrax* that were fed with feeds containing environmental microplastics and demonstrated that contaminants such as DDTs and PCBs adsorbed by the microplastics led to significant bioaccumulation in the liver.

The surface properties and porosity of microplastics, as well as the salinity, pH, and temperature of water, have a significant influence on the adsorption of heavy metals rendering microplastics carriers of chemical substances [107,112]. Presumably, metal adsorption occurs through interactions between divalent cations and oxyanions with charged or polar regions of plastic surfaces and through nonspecific interactions between neutral organometallic complexes and hydrophobic plastic surfaces [108,112]. Squadrone et al. [28] studied the presence of metals in microplastics obtained from zooplankton collected in the central-western part of the Mediterranean Sea. The highest concentrations noted were of aluminum (30 ± 2.5 mg/kg), iron (16 ± 1.9 mg/kg), and chrome (7 ± 0.008 mg/kg), but nickel (3.2 ± 0.09 mg/kg), lead (1.5 ± 0.01 mg/kg), copper (1 ± 0.01 mg/kg), and cadmium (0.033 ± 0.001 mg/kg), among other elements, were also noted on microplastics. Barboza et al. [69] reported that the occurrence of microplastics in the water substantially increased mercury concentrations in the water, and this influenced bioconcentrations in the gills and livers of European seabass (*Dicentrarchus labrax*). Simionov et al. [113] confirmed the occurrence of microplastics of the polymer polystyrene and zinc in muscle tissue of the Mediterranean mussel (*Mytilus galloprovincialis*) from the Black Sea at 37.693 mg/kg.

The widespread use of pharmaceuticals and personal care products has resulted in approximately 160 pharmaceuticals belonging to commonly used medication groups, such as antibiotics, anti-inflammatory drugs, and medicines for heart disease, being detected in surface waters and wastewater [114]. The high hydrophilicity and polarity, low octanol–water partition coefficients, and low volatility of pharmaceuticals contribute to their accumulation in the aquatic environment [108]. Li et al. [115] studied the adsorption properties of five antibiotics (sulfadiazine [SDZ], amoxicillin [AMX], tetracycline [TC], ciprofloxacin [CIP], and trimethoprim [TMP]) on five different types of microplastic particles (poly-ethylene [PE], polystyrene [PS], polypropylene [PP], polyamide [PA], polyvinyl chloride [PVC]), and reported that PA had high affinity to AMX, TC, and CIP in freshwater because of the formation of hydrogen bonds. The amounts of the five antibiotics adsorbed by PS, PE, PP, and PVC decreased as follows: CIP > AMX > TMP > SDZ > TC [115]. Nugnes et al. [116] studied the chronic and sub-chronic effects of the antiviral drug acyclovir and the insecticide imidacloprid adsorbed on polystyrene microplastics on the functioning of *Ceriodaphnia dubia*. The mixture of the selected xenobiotics inhibited reproduction and damaged the DNA of the crustacean *C. dubia* at concentrations similar to those occurring in the environment [116].

The toxicity of microplastics with adsorbed pollutants is significantly higher than the toxicity induced by microplastic particles alone, as evidenced by numerous experimental studies on animal models. The combination of microplastic with chemical contaminants (PCBs, BFRs, PFCs, and methylmercury mix) was found to result in higher hepatotoxicity in adult zebrafish compared to microplastic without adsorbed contaminants, as demonstrated by Rainieri et al. [117]. This relationship was also corroborated in the Granby et al. [118] study, wherein the investigators demonstrated that exposure of *D. labrax* to microplastic with adsorbed PCBs resulted in a notable decline in liver detoxification enzymes and interleukin β, which are implicated in the immune response and the early response to injury. No such effects were observed when the animals were fed feed with contaminants alone or microplastics alone. Furthermore, studies have concentrated on the impact of the adsorption of toxic elements. The study conducted by Barboza et al. [95] revealed that the gill and liver mercury concentrations in juvenile *D. labrax* specimens were notably higher in the presence of microplastics than in the absence of these particles. The microplastic with adsorbed contaminants contributed to the induction of oxidative stress, which in turn increased the bioconcentration of mercury in the gills and the bioaccumulation of mercury in the liver. Moreover, a study by Qin et al. [119] demonstrated that microplastics with adsorbed cadmium in the exposure of zebrafish larvae not only increased oxidative stress but also induced specific steroid hormone induction. Yu et al. [120] reported that lead-adsorbed microplastics affected the gut–hepatic and gut–brain axes, contributing to changes in gut microbiota diversity and the induction of hepatitis. The exposure of zebrafish larvae to microplastics with the adsorbed pesticide abamectin resulted in a reduction in survival, a significant increase in reactive oxygen species, impaired immune responses, and morphological changes in the eyes of the exposed organisms [121]. Furthermore, a synergistic effect of microplastics and contaminants—specified as a blend of dichlorodiphenyldichloroethylene, chlorpyrifos, and benzophenone-3—was demonstrated to elicit an inflammatory response in the distal gut of European sea bass (*D. labrax*) [122]. Montero et al. [122] observed the occurrence of lymphocyte outbreaks and abnormalities in the composition of the microflora, including a reduction in beneficial lactic acid bacteria and an increase in pathogenic bacteria such as *Vibrionales* and *Proteobacteria*. The co-occurrence of microplastics and contaminants also contributes to reproductive disorders. He et al. [123] report that triphenyl phosphate in the presence of microplastics contributed to a significant decrease in fertility rate and hatchability in zebrafish. Furthermore, the study found that the chemical stimulated gonad enlargement and inhibited spermatogenesis and oogenesis. Moreover, the presence of contaminants adsorbed by microplastics has been observed to induce behavioral changes in zebrafish. Mu et al. [124] demonstrated that co-exposure to microplastics and bisphenol analogs resulted in a reduction in movement distances and activity. Additionally, an increase in mortality of up to 51% was observed. Table 4 presents various selected contaminants adsorbed by or adhered to microplastics that can be bioaccumulated in aquatic ecosystem organisms and their potentially toxic effects on the functioning of fauna. In conclusion, co-exposure of microplastic with pollutants has been shown to increase oxidative stress in aquatic organisms and bioaccumulation of pollutants and contribute to reduced survival and reproduction.

It is noteworthy that the current state of research permits the prediction of potential effects resulting from exposure to the co-occurrence of microplastics with contaminants. However, further research is required on additional higher animal models, with a focus on chronic exposure, the use of varying concentrations of substances, and the examination of different polymers to reflect the prevailing environmental conditions.

In addition to ascertaining the dimensions, morphology, and constitution of particles ingested by organisms, it is important to employ techniques that detect the presence of additional contaminants that may have been adsorbed or utilized in industrial production, such as textile dyes. The industrial dyes present on plastic particles have the potential to enter the food chain, bioaccumulate, and cause toxicity, mutagenicity, and carcinogenicity [125]. Remy et al. [126] identified the industrial dyes Direct Blue 22 and Direct Red 28 in artificial fibers ingested by invertebrates; however, the data available are insufficient to allow an informed assessment of their toxicity.

There is also a paucity of data regarding the potential for contaminants adsorbed on microplastics to elicit further adverse effects. The current state of knowledge regarding the mechanisms of adsorption of contaminants by microplastics and their subsequent fate and transport in the presence of adsorbed contaminants remains limited. In addition, it would be essential to consider the relationship between the quantity of adsorbed contaminants, the specific type of polymer, and the prevailing environmental conditions. It is also important to emphasize that these contaminants can be transferred through seafood consumption as a secondary route of exposure, a phenomenon that has yet to be adequately studied.

**Table 4:** Types of pollutants adsorbed by microplastics that can bioaccumulate and their toxicity to aquatic organisms.

Aquatic Organisms	Pollution	Toxicity	References
Blood clam (*Tegillarca granosa*)	Bisphenol A	Neurotoxicity, immunotoxicity, increase in neurotransmitter concentration, decrease in gene expression, affecting DNA methylation	[127,128]
Copepods (*Acartia tonsa*, *Calanus finmarchicus*)	PAHs fluoranthene and phenanthrene	Bioaccumulation in lipid-rich tissues, MP-sorbed PAHs do not significantly accumulate or contribute to toxicity in marine organisms	[129]
Goldfish (*Carassius auratus*)	PAH benzo(a)pyrene	Disrupted lipid metabolism, liver damage, significantly higher Casp3 mRNA expression, oxidative stress, which leads to apoptosis	[130]
Mussel (*Mytilus coruscus*)	Dechlorane Plus	Bioaccumulation in gonads and gills, no significant effect was found	[131]
Microalgae (*Chlorella vulgaris*)	Dechlorane Plus	Reduced photosynthetic efficiency (reduced Fv/Fm by 0.03%), higher growth inhibition (16.15%) and oxidative damage (increased ROS by 152%), co-exposure significantly downregulated amino acid metabolism and tricarboxylic acid cycle (TCA) cycle and upregulated fatty acid metabolism	[132]
Atlantic cod (*Gadus morhua*)	PCB-126	Bioaccumulation in livers and muscles, minor differences in the cyp1a expression in liver and skin histology	[133]
European seabass (*Dicentrarchus labrax*)	DDE, BP-3, chlorpyrifos	Bioaccumulation in livers and muscles, no effect on fish condition indicators was observed	[111]
Blue discus (*Symphysodon aequifasciatus*)	Cadmium	Oxidative stress, stimulation of innate immunity in young individuals, antagonistic interaction between the two stressors (MP and cadmium)	[134]
Crucian carp (*Carassius carassius*)	Cadmium	Inflammation of liver and spleen cells, reduction in the diversity and number of intestinal microflora organisms, oxidative stress, a significant upregulation in the gene expression levels of il-8 and hsp70	[135]
*Daphnia magna*	Cadmium	Microplastic and Cd has additive effects on feeding and growth rates, resulting in a greater energy allocation shift	[136]
Microalgae (*Microcystis aeruginosa*)	Arsenic	oxidative stress, fatty acid metabolism was significantly upregulated	[137]
Clam (*Ruditapes* *Philippinarum*)	Mercury	Decreased filtration rates, gill and digestive gland pathology, immunotoxicity, oxidative stress biomarkers remained unchanged	[138]

In future research, the long-term effects of exposure to contaminants and/or MPs in fish should be determined to understand the effects on the health and robustness of the fish later in life. The interactions between MPs and contaminants and their combined effects on fish health are far from understood and need more research.

## 6. Human Health Impacts

Humans are exposed to microplastics because these particles are dispersed readily throughout the biosphere; exposure comes through the consumption of contaminated foods, drinking water, or air [139,140]. Humans are usually exposed to microplastics through the consumption of fishes and mussels. Most evidence indicates that these contaminants accumulate in the gastrointestinal tract, but, according to reports, removing the gastrointestinal tract from fish could not eliminate the risk of humans’ exposure to microplastics [73,141]. Further, fishmeal is used as a basic ingredient in formulated fish feed in aquaculture and animal husbandry, which is why microplastics can also come from other sources of marine food [141,142]. Some studies suggest that aquatic microplastics may act as vectors of microbiological toxicity, carrying biofilm-associated opportunistic bacterial pathogens and antibiotic resistance genes that may interact with gut microbiota, and they can also be carriers of fungi and viruses [143].

Microplastic particles can damage the lungs and intestines, and especially small particles can penetrate cell membranes, the blood–brain barrier, and the human placenta [144]. Inhaled microplastics, due to their small sizes, can translocate into the respiratory epithelium via diffusion, direct cellular penetration, or active cellular uptake [140]. Upon entering the respiratory system, microplastics reach the alveoli, contributing to inflammation, oxidative stress, and lung dysfunction including asthma, pneumonia, allergic reactions, and deformations of bronchial tissues [145]. Baeza-Martínez et al. [146] conducted a study aimed at identifying the occurrence of microplastics in the lower respiratory tracts of Europeans from whom bronchoalveolar lavage fluid (BALF) was sampled. They concluded that most of the microplastics identified were rayon and polyester microfibers at a mean concentration of 9.18 ± 2.45 items/100 mL BALF. Further, they identified a dependence between smoking and the concentration of microplastics in BALF. The occurrence of microplastic particles, particularly in the synthetic textile industry, poses significant occupational risks to workers through constant exposure. Inhalation of plastic fibers and particles, especially by exposed workers, has been demonstrated to present as shortness of breath caused by inflammatory reactions in the airways and interstitial pneumonia [143,147]. In a study conducted on rats, Wang et al. [107] describe nephrotoxicity caused by exposure to microplastics and indicate damage to kidney tissue, including glomerular division, inflammatory infiltration, missing brush border, and detachment of renal tubular epithelial cells, and also renal interstitial hemorrhage. Recently, Massardo et al. [148] reported the presence of microplastics in kidneys based on analyses of kidney samples collected during nephrectomies and also in urine from healthy subjects. They discovered 26 microplastic particles ranging in size from 3 to 13 µm in urine and from 1 to 29 µm in kidneys. The particles were identified as polyethylene, which, among other applications, is used widely for the production of bottles and food packaging. Further, these authors report the occurrence of the pigments hematite and copper phthalocyanine. These pigments are possible additives for polymers, used to reduce their costs, acting as fillers, but also to improve performances in terms of stability, durability, electrical resistance [148]. A study Ragusa et al. [149] conducted revealed the occurrence of pigmented microplastic particles in the placentas of female subjects, which raises serious concerns. Microplastic can penetrate the bloodstream and placenta from the respiratory or gastrointestinal tract of pregnant women [144]. These researchers demonstrated that microplastics could cause changes in immune response mechanisms during pregnancy, which could lead to preeclampsia and impaired fetal growth. Further, in another study, Ragusa et al. [150] investigated breast milk, of which 26 of 34 samples contained microplastic particles of polyethylene, polyvinyl chloride, and polypropylene ranging in size from 2 to 12 µm. Milk is a favorable environment for the lipophilic nature of microplastics because it consists of protein and fat globules in a carbohydrate-based suspension [149]. In a study of infant formulas, Kadac-Czapska et al. [144] detected microplastics in all 30 products tested. Based on the manufacturers’ recommended formula portions, the estimated daily intake of microplastics by infants from birth to six months was 49 ± 32 microplastic particles/daily. Xu et al. [151] report that infants might be exposed to microplastics from feeding bottles. In their study, these researchers showed that microplastic particles are released by physical factors such as abrasion or thermal–oxidative aging process during brewing milk powder preparation, boiling water disinfection, and microwave heating. Further, this study indicated that microplastic exposure induced oxidative stress and inflammation in human intestinal cells. Considering the necessity of providing infants with complementary foods from the sixth month of life, the number of microplastics consumed will increase because children drink formula even up to the age of three years, while simultaneously drinking water from feeding bottles and consuming food often packaged in bags or tubes, which can be another source of plastic [144].

Human health risks associated with microplastic exposure include the occurrence of inflammation, oxidative stress, and DNA damage, which can lead to diseases of the circulatory and respiratory systems and also cancers. Studies that detect microplastics in the human body are raising concerns in scientific communities since knowledge on the topic of human exposure to them remains very limited, and the full impact of microplastics on human health is not yet well understood [145,152].

## 7. Conclusions and Future Research

This manuscript presents a critical analysis of the pervasive problem of microplastics in the aquatic environment. A comprehensive review of the presence of microplastics in sediments and water reservoirs was conducted, and numerous studies were cited indicating the presence and toxicity of microplastics to aquatic organisms and humans. Potential directions for future research are also presented. Despite the rich literature on the subject, many questions still remain unanswered.

The latest research results are disturbing; they prove that microplastics have the ability to penetrate all tissues and internal organs, e.g., the liver, kidneys, lungs, intestines, and reproductive organs, and may disturb their functions. However, the lack of comprehensive data makes it difficult to determine the potential health effects of acute and especially chronic exposure. In the context of the health of future generations, the presence of microplastics in the placenta and mother’s milk is also worrying.

Another significant problem is the adsorption of a number of pollutants on microplastic particles. Therefore, a more detailed analysis of the mechanisms of adsorption of pollutants by microplastics is necessary, as well as an assessment of the long-term exposure of living organisms. It is important to understand the impact of complex pollutants on aquatic systems, which provides an important scientific basis for environmental protection and sustainable development.

The processes of bioaccumulation and biomagnification are complex. Not only the adsorption/desorption capacity of MPs, the type of polymer, and MP degradation processes, but also the exposure time of organisms, the efficiency of digestive processes, the ability to metabolize toxic substances, etc., are important. This makes it often difficult to obtain unambiguous results.

Therefore, it is necessary to carry out long-term experiments using different species with concentrations of MPs and toxic pollutants similar to those in the environment and in conditions as realistic as possible. In addition, further research on the adsorption of toxic compounds on MP particles, their trophic transfer, and exposure to living organisms will be necessary.

The above studies should also take into account the influence of environmental factors such as temperature, pH, salinity, UV radiation, and hydrodynamic conditions.

It is also important to pay attention to the development of quick and effective methods for identifying types of microplastics. This will enable the introduction of actions aimed at reducing the burden on the aquatic environment.

Due to the intensive development of industry and the use of plastics in almost all areas of life, it is impossible to completely eliminate this problem. It is therefore important to pay attention to the implementation of legal regulations regarding not only limiting the use of plastics, but also determining limits, e.g., in the form of MRLs, in food raw materials, especially those of aquatic origin.

## Figures and Tables

**Table 1:** Occurrence of microplastics in littoral, surface, and beach sediment samples.

Location	Number of Particles/kg d.m.	Particle Size	Types of Polymers	References
Persian Gulf, Iran, littoral sediments	61 ± 49	10 µm–4.7 mm	PE, PET, NY	[43]
Northern Bering and Chukchi Seas, surface sediments	5.3–68.9	0.1–4.7 mm	PP, PET, RY	[44]
Western Mediterranean continental shelf, Spain, surface sediments	113.2 ± 88.9	<0.5 mm	PS, PA, PMMA	[45]
Haichow Bay, central coast, China, beach sediments	106.50 ± 34.41	0.01–5 mm	PE, PP, PS, PET, nylon	[46]
Virginia and North Carolina, USA, beach sediments	1410 ± 810	0.5–5 mm	PE, PP, PVB, PET, PTFE, PS	[47]
Southeast coast, Bangladesh, beach sediments	242.86	0.2–5 mm	PE, PP, PS, PU, PET, PVC	[48]
South Andaman beaches, India, sediments	414.35 ± 87.4	500–1000 µm	PP, PVC, PS, PBR	[49]
Dubai coast, UAE, beach sediments	59.71	-	PE, PP	[50]
Northern Oman Sea, litorral sediments	138.3 ± 4.5–930.3 ± 49.1	100–5000 µm	PE, PP, NY	[51]
Wonorejo coast, Surabaya, Indonesia, sediments	590	-	LDPE, PE, PP	[52]
Rivers of the Tibet Plateau, China, sediment	50–195	<1 mm	PET, PE, PP, PS, PA	[53]
Chao Phraya River, Thailand, sediment	2290	0.053–0.5 mm	PP, PE, PS	[54]
North Yellow Sea, China, sediments	37.1 ± 42.7	<1 mm	PP	[55]
Pearl River along Guangzhou City, China, sediments	80–9597	-	PP, PE	[56]
Coastal plain river network in eastern China, sediments	32,947 ± 15,342	<300 μm	PP, PE, PS	[57]

**Notes:** PE—polyester; PET—polyethylene terephthalate; NY—nylon; PP—polypropylene; RY—rayon; PS—polystyrene; PA—polyamide; PMMA—poly(methyl methacrylate); PVB—poly(4-vinylbiphenyl); PTFE—polytetrafluoroethylene; PU—polyurethane; PVC—polyvinyl chloride; PBR—polybutadiene rubber; LDPE—low-density polyethylene.

**Table 2:** Occurrence of microplastics in surface water samples.

Location	Number of Particles/m^3^	Particle Size	Types of Polymers	References
Rivers of the Tibet Plateau, China	483–967	<1 mm	PET, PE, PP, PS, PA	[53]
Chao Phraya River, Thailand	104–805.2	0.5–1 mm	PP, PE, PS, PTFE, EVA, cellophane	[54]
Ofanto River, Italy	0.9 ± 0.4–13 ± 5	300–5000 µm	PE, PP, PS, PVC, PUR	[58]
Rawal Lake, Pakistan	6.4–8.8 ± 0.5	0.1–5 mm	PE, PP, PS	[59]
Ganges River, India	92.85 ± 50.69	100–2000 µm	PET, PA, PE, PP, PVC, PS	[60]
Rivers flowing into the southern Caspian Sea, Iran	0.407–1.406	≤1 mm	PE, PS, PET	[24]
Pearl River along Guangzhou City, China	379–7924	-	PP, PE	[56]
Crater lake in Erzurum, Turkey	-	8–15 μm	PP, PE	[61]
Ox-Bow Lake, Nigeria	Dry season: 1004–8329 Raining season: 201–8369	-	Dry season: PET, PVC Raining season: PVC, LDPE, PE, PET, PA, PES	[62]
Lake Ontario, Canada	0.8 particles/L	-	PET, PE, PVC, cellulose	[63]
North Yellow Sea	545 ± 282	<1 mm	PE	[55]
Deep Bay, Tolo Harbor, Tsing Yi, and Victoria Harbor, China	51–27,909 particles/100 m^3^	0.03–4.96 mm	PP, LDPE, HDPE, EPDM, SAN	[64]

**Notes:** PET—polyethylene terephthalate; PE—polyester; PP—polypropylene; PS—polystyrene; PA—polyamide; PTFE—polytetrafluoroethylene; EVA—ethylene-vinyl acetate; PVC—polyvinyl chloride; PUR—polyurethane; LDPE—low-density polyethylene; PES—polyethersulfone; HDPE—high-density polyethylene; EPDM—polypropylene/ethylene-propylene-diene monomer; SAN—styrene acrylonitrile.

**Table 3:** Microplastics found in organisms that live in freshwater and seawater.

Freshwater				
Aquatic Organism	Types of Plastics	Particle Size	Location	References
Biota (Nile Tilapia)	PE, PET, PP	7.5 ± 4.9 (items/organism)	Egypt	[71]
Biota (riverine fish—guts)	PA, PE, PS	8.12 ± 4.26 (items/organism)	Iran	[72,73]
Biota (fish)	PS, PE, PA	4.20 ± 3.32–12 ± 11.31 (items/organism)	Iran	[74]
Goldfish (*Carassius* *auratus*)	Microbeads microfibers	3 particles/50 retained	Canada	[75]
Gerreidae fish (*Eugerres brasilianus*, *Eucinostomus melanopterus* and *Diapterus rhombeus*)	Blue nylon fragments	4.9 and 33.4% of individuals	Tropical estuary in Northeast Brazil	[76]
European flounder (*Platichtyhys flesu*)	NY, PA, PE	Concentration of MP fibers in the gut 75%	River Thames, UK	[77]
European smelt (*Osmerus eperlerus*)	NY, PA, PE	Concentration of MP fibers in the gut 20%	River Thames, UK	[77]
Crayfish (*Procambarus clarkii*)	Fiber and fragments	0.17 ± 0.07–0.92 ± 0.19 (particles/individual)	China	[78]
*Squalius cephalus*	Fiber and fragments	2.41 mm	France	[79]
Bluegill (*Lepomis* *Macrochirus*) and Longear (*Lepomis* *Megalotis*)	NY, PA, PE	Concentration of MPs 45%	Brazos River Basin, USA	[80]
**Seawater**				
**Aquatic Organism**	**Types of Plastics**	**Particle Size**	**Location**	**References**
*Rutilus frisii kutum*	Fragments, fibers, Beads	11.4 ± 1.68 (items/organism)	Iran (Caspian Sea)	[81]
*Periophthalmus waltoni*	PS, PE, PET, PA	15 (items/organism)	Iran (Arab/Persian Gulf)	[82]
*Siganus rivulatus*	Fragments, fibers	59.7 (items/individual)	Israel (Mediterranean Sea)	[83]
Brown shrimp (*Crangon* *crangon*)	-	1.23 ± 0.99 (items/ individual)	Southern North Sea (UK, France, Belgium, and The Netherlands)	[84]
Crustacea (*Euphausia* *pacifica*)	-	0.059 (items/ individual)	Northeast Pacific Ocean, Canada	[85]
Bivalvia (*Alectryonella* *plicatula*)	-	4.3–57.2 (items/ individual)	China	[86]
Bivalvia (*Mytilus edulis*)	-	34–178 (items/ individual)	Halifax Harbor, Canada	[87]
Bivalvia (*Mytilus edulis*)	-	1.1–6.4 (items/ individual)	Coastal waters of the United Kingdom	[88]
Gastropoda (*Cerithidea* *cingulata*)	-	17.7 ± 0.3 (items/ individual)	Iran (Persian Gulf)	[89]
Gastropoda (*Colus* *jeffreysianus*)	-	0.678 ± 0.044 (items/ individual)	Rockall Trough, North Atlantic Ocean	[90]

**Notes:** PE—polyester; PET—polyethylene terephthalate; PP—polypropylene; PA—polyamide; PS—polystyrene; NY—nylon.

## Data Availability

Data available on reasonable request.
